# Clinical evaluation of an allogeneic bone matrix containing viable osteogenic cells in patients undergoing one- and two-level posterolateral lumbar arthrodesis with decompressive laminectomy

**DOI:** 10.1186/s13018-016-0392-z

**Published:** 2016-05-27

**Authors:** David B. Musante, Michael E. Firtha, Brent L. Atkinson, Rebekah Hahn, James T. Ryaby, Raymond J. Linovitz

**Affiliations:** Triangle Orthopedics, 120 William Penn Plaza, Durham, NC 27704 USA; Campbell School of Osteopathic Medicine, Campbell University, 4350 US-421, Lillington, NC 27546 USA; Atkinson Biologics Consulting, 9189 Fox Fire Way, Highlands Ranch, Littleton, CO 80129 USA; Orthofix Inc., 3451 Plano Parkway, Lewisville, TX 75056 USA

**Keywords:** Cellular bone allograft, Spinal arthrodesis, Bone graft extender, Trinity Evolution, Lumbar spine fusion

## Abstract

**Background:**

Trinity Evolution® cellular bone allograft (TE) possesses the osteogenic, osteoinductive, and osteoconductive elements essential for bone healing. The purpose of this study is to evaluate the radiographic and clinical outcomes when TE is used as a graft extender in combination with locally derived bone in one- and two-level instrumented lumbar posterolateral arthrodeses.

**Methods:**

In this retrospective evaluation, a consecutive series of subject charts that had posterolateral arthrodesis with TE and a 12-month radiographic follow-up were evaluated. All subjects were diagnosed with degenerative disc disease, radiculopathy, stenosis, and decreased disc height. At 2 weeks and at 3 and 12 months, plain radiographs were performed and the subject’s back and leg pain (VAS) was recorded. An evaluation of fusion status was performed at 12 months.

**Results:**

The population consisted of 43 subjects and 47 arthrodeses. At 12 months, a fusion rate of 90.7 % of subjects and 89.4 % of surgical levels was observed. High-risk subjects (e.g., diabetes, tobacco use, etc.) had fusion rates comparable to normal patients. Compared with the preoperative leg or back pain level, the postoperative pain levels were significantly (*p* < 0.0001) improved at every time point. There were no adverse events attributable to TE.

**Conclusions:**

Fusion rates using TE were higher than or comparable to fusion rates with autologous iliac crest bone graft that have been reported in the recent literature for posterolateral fusion procedures, and TE fusion rates were not adversely affected by several high-risk patient factors. The positive results provide confidence that TE can safely replace autologous iliac crest bone graft when used as a bone graft extender in combination with locally derived bone in the setting of posterolateral lumbar arthrodesis in patients with or without risk factors for compromised bone healing.

**Trial registration:**

Because of the retrospective nature of this study, the trial was not registered.

## Background

Lumbar arthrodesis is a commonly performed surgical procedure in the treatment of numerous spinal diagnoses including degenerative disc disease, spinal stenosis, spondylolisthesis, and other deformities. A bony fusion is essential for restoring segmental stability, preventing or correcting deformity and, when combined with decompression for disorders such as spondylolisthesis with spinal stenosis, has been shown to provide improved long-term outcomes [1, 2]. Lumbar intervertebral fusion is achieved by creating an environment conducive to the formation of a continuous osseous bridge across the involved spinal segments. Autologous iliac crest bone graft (ICBG) has historically been the gold standard bone grafting material used to create this fusion environment because of its inherent biological characteristics of osteoconduction (scaffold), osteoinduction (signal), and osteogenesis (viable cells). However, harvesting of ICBG requires a second operative site which is associated with complications such as chronic harvest site pain, infection, increased operative time, and blood loss [3–7]. Additionally, the quality and quantity of ICBG may be inadequate especially in older individuals or patients with significant comorbidities [8, 9].

To counter these issues, a plethora of bone graft substitutes (BGS) have been developed such as freeze-dried bone allograft, demineralized bone matrices (DBM), synthetic matrices, and recombinant bone morphogenetic protein (BMP). An ideal BGS should be safe, economical, and comparable in composition to ICBG so as to contain a physiologic quantity and quality of viable osteoprogenitor cells, matrix, and signal. However, until recently, none of the available BGS contained all three components (cells, signal, matrix) in a single “off the shelf” tissue form.

Trinity Evolution® cellular bone allograft (TE) is a viable, cryopreserved cellular bone allograft. Cellular bone allografts consist of viable cancellous bone and demineralized cortical bone and contain physiologic amounts of living, healthy osteogenic cells (such as mesenchymal stem cells and osteoprogenitor cells), signals (bioactive BMP endogenous to the demineralized cortical bone), and a scaffold (cancellous matrix) to which the progenitor cells remain attached [10]. TE is regulated by the FDA to be a human cell and tissue product (HCT/P) and is intended for use in bone repair, replacement, or reconstruction. Due to possessing biologic properties that closely resemble those of autograft, TE may be well-suited for usage in patients with comorbidities such as advancing age, long-term steroid therapy, and cigarette smoking, which may compromise the quality of autograft and are known to have a negative effect on bone healing [11]. Previously, TE was evaluated in foot and ankle arthrodesis procedures in patients with comorbidities and fusion rates were higher than or comparable to fusion rates with autograft that were reported in the literature [12].

To evaluate the clinical results of TE, we conducted a restrospective study with plain radiographic evaluation of a population of subjects requiring posterolateral lumbar arthrodeses. The purpose of this clinical study was to review the radiographic and clinical outcomes when TE is used as a graft extender with locally derived autograft bone. To our knowledge, this is the first clinical report of a cellular bone allograft used in posterolateral lumbar arthrodesis without interbody arthrodesis.

## Methods

### Study design

In this retrospective evaluation, a consecutive series of charts in which the subjects had surgery at a single institution after June 2009 and who completed their 12-month visit before October 28, 2014 were screened to determine whether they met the inclusion criteria. These criteria included being at least 18 years of age and receiving posterolateral lumbar (L1-S1) fusion surgery with decompressive laminectomy that utilized TE that was mixed with locally derived autograft bone in a one- or two-level lumbar arthrodesis procedure with supplemental internal pedicle screw fixation. Patients were excluded who required more than two-level arthrodeses and interbody fusions, did not return for a follow-up visit within the 12 ± 3-month window, or who did not have flexion/extension films at the 12-month visit. No restrictions were placed on the diagnosis, which included degenerative disc disease, spondylolisthesis (all grades), scoliosis, radiculopathy, reflex changes, stenosis, instability, osteophytes, decreased disc height, herniated nucleus pulposus, facet joint degeneration, and/or vacuum phenomenon. The Western Institutional Review Board approved this study (number 1150670) and waived the requirement to obtain consent.

### Surgical procedure

All patients underwent open decompressive laminectomy, medial facetectomy, and posterolateral arthrodesis with pedicle screw instrumentation with intraoperative fluoroscopic assistance.

Decompressive laminectomy was performed by removing approximately the inferior two thirds of the cephalad spinous process and lamina and approximately the superior half of the caudal spinous process and lamina and intervening ligaments extending laterally to include medial facetectomy. Complete facetectomy was avoided. Foraminotomies were performed when necessary. The preparation of TE (Orthofix, Inc., Lewisville, TX, processed by Musculoskeletal Transplant Foundation, Edison, NJ) in the operating room prior to implantation was standardized in accordance with the instructions for use as previously described [13]. All locally harvested bone was obtained from resected spinous processes, laminae, and medial facets and stripped of soft tissue and morselized. The volume was reliably measured in a graduated, clear medicine cup prior to mixing with TE.

Meticulous decortication of the transverse processes, the lateral facet joint, and the facet joint itself was performed with a power burr. The combined morselized local autograft and TE was packed across the decorticated transverse processes and intertransverse space and into the facet joints bilaterally.

### Postoperative management and data collection

The charts were reviewed for a total of three postoperative visits that occurred at 2 weeks and at 3 and 12 months. At each time point, the visual analogue scale (VAS) for leg and back pain, adverse reactions, and plain radiographs were collected. During surgery, the volumes of local bone and TE implanted and the volume of blood loss were recorded. At the 12-month visit, four lumbar plain radiographic views (anterior-posterior, neutral lateral, and lateral flexion and extension) were obtained using a digital system.

The chart review showed that pulsed electromagnetic field (PEMF) stimulation (Spinal Stim®; Orthofix, Lewisville, TX) was prescribed for all four subjects that received a two-level arthrodesis and in two subjects that received a one-level procedure.

Patient age, gender, body mass index (BMI), race, working status, diagnosis, and comorbidities (nicotine, diabetes, osteoporosis, and steroid dependence) were collected.

### Radiographic evaluation

After a subject met inclusion/exclusion criteria and was included in the study, the investigator, who was not blinded to the treatment group, performed an evaluation of the 12-month plain radiographs stored on a PACS (SECTRA IDS7) and viewed on a high-definition monitor. Prior to study onset, a fused criterion was defined which required both bridging bone (unilateral or bilateral bridging bone would be considered fused) and angular vertebral motion less than or equal to 4° on flexion/extension lateral plain radiographs. The investigator evaluated the anterior/posterior (A/P) radiograph and denoted either “yes” or “no” for bridging bone and also denoted which side was bridged or whether bridging was bilateral. For motion determination, the Cobb angles between the superior endplates of each treated level on flexion and extension radiographs were measured using the PACS software and the difference calculated. In two-level procedures, both levels had to be fused for the subject to be classified as fused.

### Statistical methods

Student’s *t* test was performed to compare the mean back or leg pain at each follow-up time to the baseline values. Student’s *t* test was also used to determine if the mean volumes of TE and autograft bone varied between subjects who fused and subjects who did not fuse.

Fisher’s exact test was utilized to evaluate the subgroups stratified among various treatment, risk, or demographic factors for the potential association with fusion outcome. SAS 9.4 software was used for all statistical evaluations.

Data are reported as mean ± SD, and the significance levels for all tests were set at a *p* value of less than 0.05.

## Results

Forty-three subject charts were enrolled in the study, and arthrodesis was performed at 47 levels.

### Baseline characteristics

Of the 43 subjects, 63 % were female and 37 % were male with the mean age of 64.50 ± 10.5 years (age range, 44 to 85). The demographics, comorbidities, and diagnoses varied across the patient population (Tables 1, 2, and 3). All subjects were diagnosed with degenerative disc disease (DDD) and its sequelae such as radiculopathy, stenosis, and decreased disc height. Twenty-five percent of the surgical population was diagnosed with scoliosis. Ninety-five percent were diagnosed with spondylolisthesis (78 % grade I, 22 % grade II). Eighty-eight percent of the subjects were overweight, obese, or morbidly obese (BMI at least 25 to more than 40), 9 % of the subjects were non-insulin-dependent diabetics, and 9 % were smokers. Thirty-nine subjects (90.7 %) received fusion at one level and four (9.3 %) received fusion at two levels. Arthrodeses were performed 7 times at L3-L4 and 40 times at L4-L5. No lumbar-sacral fusions were performed. No complications were reported intraoperatively. Postoperative blood transfusion was performed in 4.6 % of the patients.

**Table 1:** Demographic frequency

Demographic		*n*	(%)
Age			
	<65	21	(48.84)
	65+	22	(51.16)
Gender			
	Female	27	(62.79)
	Male	16	(37.21)
Weight status			
	Underweight (<18.5)	0	(0)
	Normal weight (18.5-24.99)	5	(11.63)
	Overweight (25–29.99)	11	(25.58)
	Obese (30–39.99)	24	(55.81)
	Morbidly obese (≥40)	3	(6.98)
Race			
	Unknown/undisclosed	1	(2.33)
	Black or African American	8	(18.60)
	White	34	(79.07)
Working status			
	Workers compensation	1	(2.33)
	Disability^a^	5	(14.71)

^a^Only 34 subjects had information on disability status; workers compensation, and disability do not overlap

**Table 2:** Surgical factor frequency

Surgical factor	*n*	(%)
Number of levels		
One	39	(90.70)
Two	4	(9.30)
Levels^a^		
L3–L4	7	(14.89)
L4–L5	40	(85.11)
Blood transfusions		
No	41	(95.35)
Yes	2	(4.65)
Diagnosis^b^		
DDD	43	(100)
Spondylolisthesis	41	(95.35)
Grade I	32	(78.05)
Grade II	9	(21.95)
Scoliosis	11	(25.58)
Radiculopathy	43	(100)
Reflex changes	13	(30.23)
Stenosis	43	(100)
Instability	41	(95.35)
Osteophytes	8	(18.60)
Decreased disc height	43	(100)
Herniated nucleus pulposis	5	(11.63)
Facet joint degenration	41	(95.35)
Vacuum phenomenon	0	(0)
Other (facet cyst)	4	(9.31)
Local bone volume		
<10 cc	8	(18.6)
10+ cc	35	(81.4)
PEMF^c^		
No	37	(86.05)
Yes	6	(13.95)

^a^There were a total of 47 levels treated

^b^All relevant diagnoses could be selected for each subject

^c^Pulsed electromagnetic field stimulation

**Table 3:** Risk factor frequency

Risk factors	*n*	(%)
Tobacco users		
No	39	(90.70)
Yes	4	(9.30)
Diabetes		
No	34	(79.07)
Yes^a^	9	(20.93)
Osteoporosis		
No	40	(93.02)
Yes	3	(6.98)
Steroid dependence		
No	38	(88.37)
Yes	5	(11.63)

^a^None of the subjects with diabetes were insulin dependent

### Fusion

**Fig. 1:**
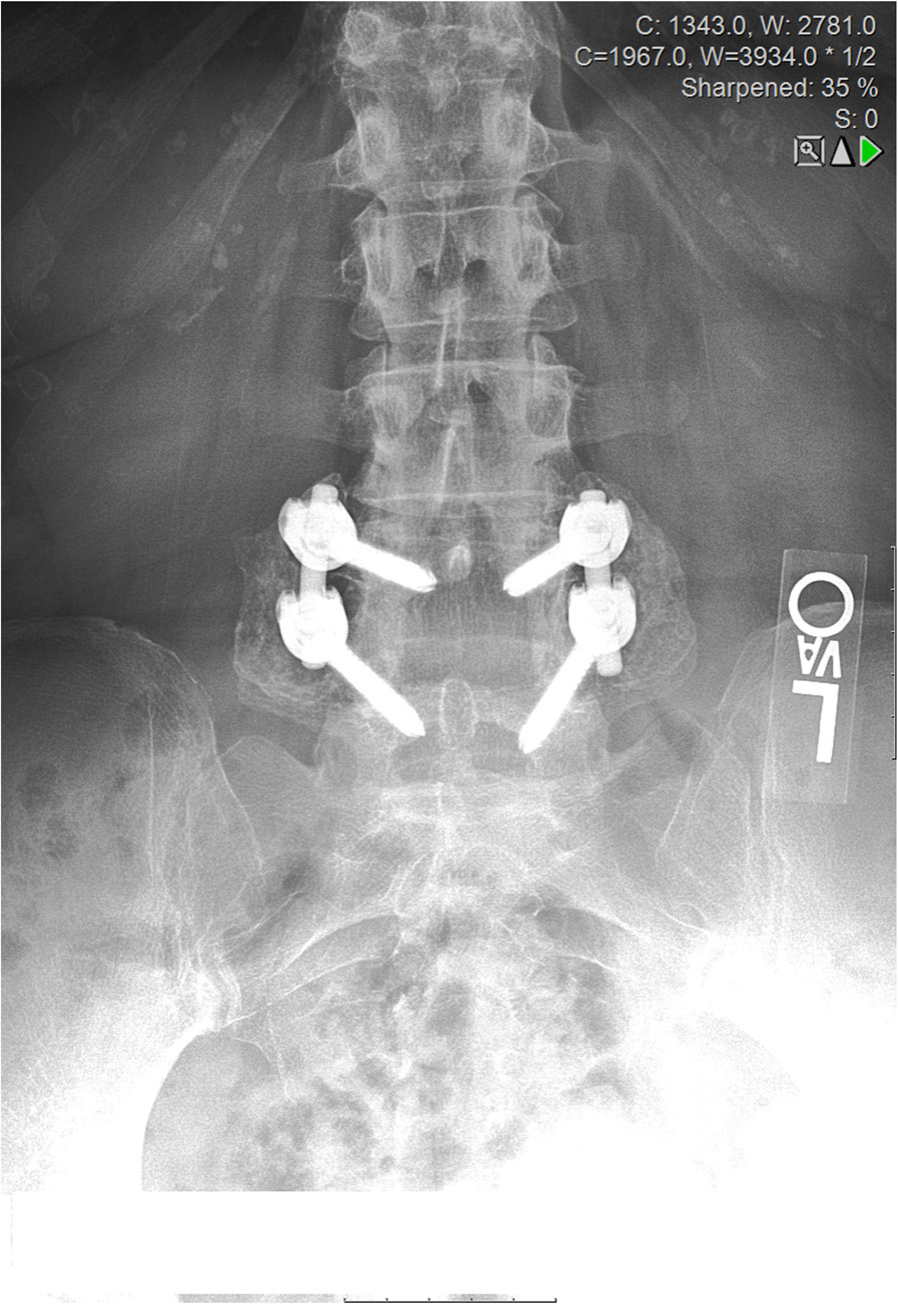
Twelve-month visit AP radiograph. Subject that was diagnosed with grade II degenerative spondylolisthesis and osteoporosis and that was treated with osteoporosis-related medications. Subject received 15 cc TE and 10 cc locally derived autograft. Robust bilateral bone bridging observed at 10.5 months. Patient fell at 6 months postoperatively and obtained a T9 compression fracture, although this apparently had no adverse effect on the fusion mass

**Fig. 2:**
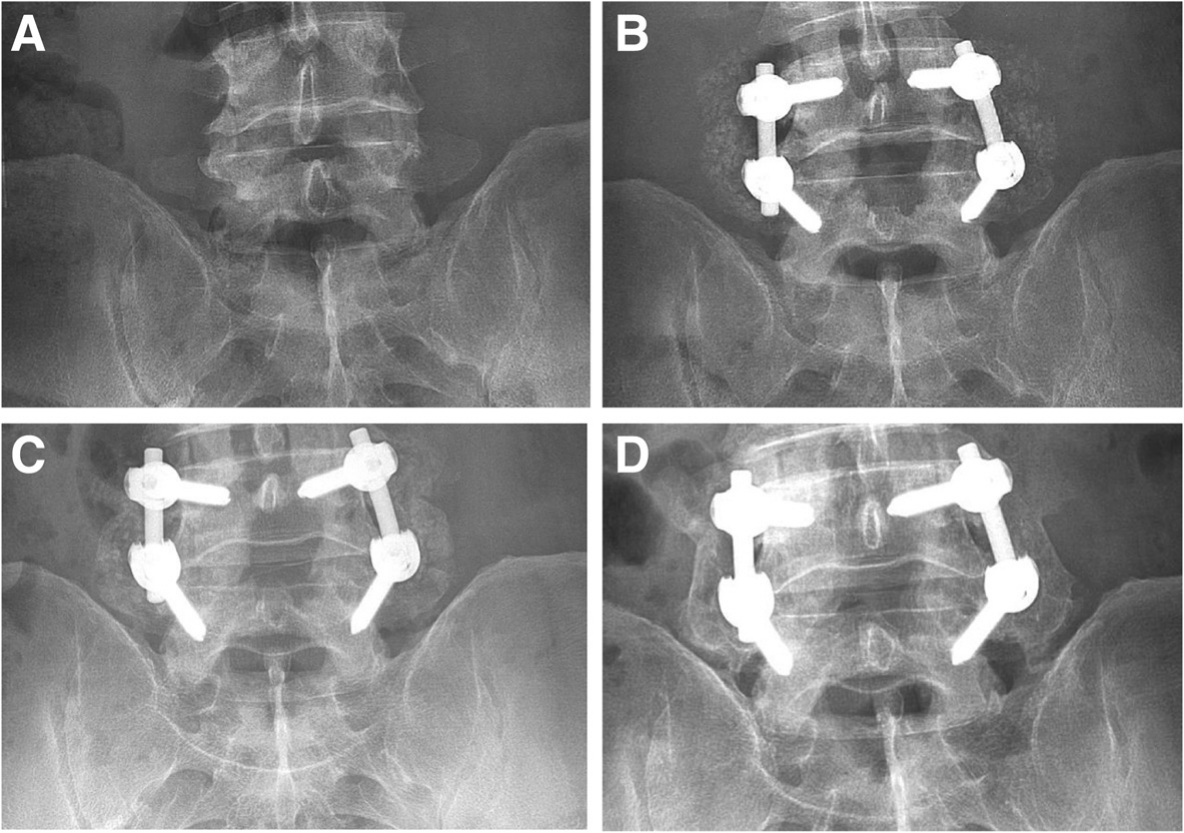
Baseline, 2-week, and 2- and 12-month AP radiographs. These radiographs show maturation of robust bilateral bridging bone over time. Subject was a non-insulin-dependent diabetic patient with grade I degenerative spondylolisthesis. Subject received 10 cc of TE and 15 cc of locally-derived autograft. **a** Baseline. **b** 2 weeks. **c** 2 months. **d** 12 months

At 12 months, a fusion rate of 90.7 % of the subjects and 89.4 % of surgical levels was observed (Table 4, Figs. 1 and 2) using the radiographic criteria established prior to the study. Facet fusion could not be assessed from plain radiographs, but may have contributed to decreased motion as indicated in one subject that had less than 4° of motion, but no bridging bone. The incidence of bridging bone was not significantly different between the two levels (85.7 % for L3-L4 and 87.5 % for L4-L5). In some cases, robust bridging bone was observed as early as 3 months (Fig. 2).

**Table 4:** Fusion status at 12 months

Fusion at 12 months	Per subject		Per level	
	*n*	(%)	*n*	(%)
Fused^a^	39	(90.7)	42	(89.4)
Not fused	4	(9.3)	5	(10.6)

^a^Fused requires both bridging bone and angular vertebral motion ≤4° on flexion/extension lateral plain radiographs per level and both levels to be fused per subject. Fusion was analyzed post hoc

Three subjects did not meet the fused criteria but were asymptomatic. One subject received a two-level arthrodesis that showed signs of bridging bone at 3 months before the patient had a falling accident. The other two subjects received a single-level arthrodesis.

The mean volume of TE or autograft was similar and not statistically different in subjects that successfully fused compared to subjects that did not fuse (Table 5). Per arthrodesis level, the TE volume mean was 12 ± 2.5 cc (range of 10 to 15 cc). The local bone volume mean was 11.1 ± 2.7 cc (range of 5 to 20 cc). The mean total volume of the combined graft was 22.6 ± 4.0 cc (range for one-level procedures was 15–30 cc and the range for two-level procedures was 30–45 cc). Fusion was observed in two patients that had a low volume of autograft bone (5 and 8 cc) that contributed to the volume mass.

**Table 5:** Fusion status at 12 months stratified on various treatment, risk, or demographic factors

Demographic		% fused	(#/*n*)
Weight status			
	Normal weight (18.5–24.99)	100	(5/5)
	Overweight (25–29.99)	81.8	(9/11)
	Obese (30–39.99)	94.1	(16/17)
	Morbidly obese (≥40)	90.0	(9/10)
	*p* value	0.73	
Diabetes			
	No	91.2	(31/34)
	Yes	88.9	(8/9)
	*p* value	1	
Tobacco users			
	Not currently	92.3	(36/39)
	Currently	75.0	(3/4)
	*p* value	0.33	
Gender			
	Female	88.9	(24/27)
	Male	93.8	(15/16)
	*p* value	1	
Age			
	<65	85.7	(18/21)
	65+	95.5	(21/22)
	*p* value	0.34	
Number of levels			
	One	92.3	(36/39)
	Two	75.0	(3/4)
	*p* value	0.33	
Local bone volume			
<10 cc per level		75.0	(6/8)
10+ cc per level		94.3	(33/35)
*p* value		0.13	
PEMF			
No		91.9	(34/37)
Yes		83.3	(5/6)
*p* value		0.46	
Steroid dependence			
	No	89.5	(34/38)
	Yes	100	(5/5)
	*p* value	1	

The subgroups were stratified among various diagnoses, risk, and demographic factors and evaluated for the potential association with fusion outcome. With the numbers provided, there were no significant differences detected in fusion rates among subjects who were normal weight as compared to subjects who were overweight, obese, or morbidly obese; were diabetic as compared to those who were not; had never used or who were former or current tobacco users; were male or female; were over or under the age of 65; received a two-level or a one-level arthrodesis; received or did not receive bone growth stimulation; or were or were not steroid dependent (Table 5).

### Clinical findings

**Fig. 3:**
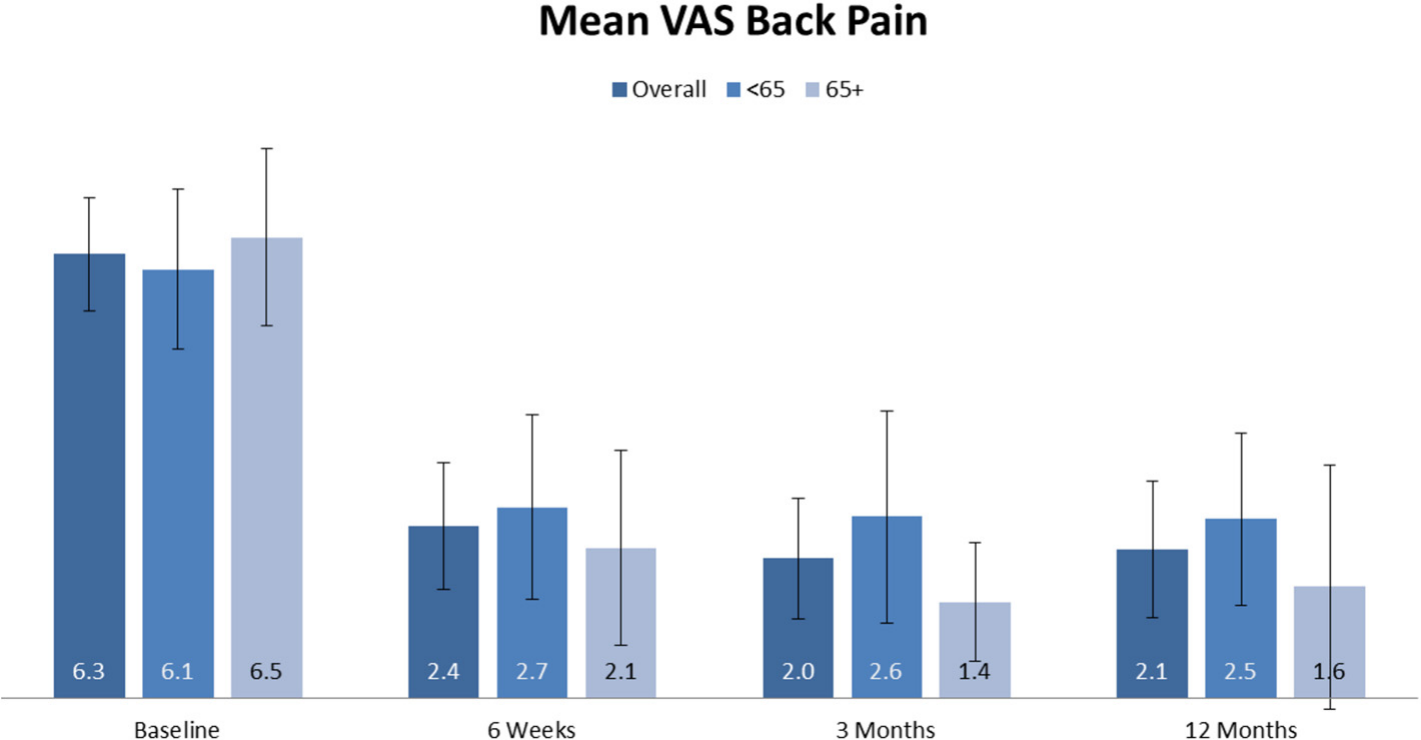
Back VAS pain scores at baseline, 2 weeks, and at 3 and 12 months. At each time-point, the *bars* from *left* to *right* indicate scores for the entire population, subjects aged less than 65 years old, and those 65 years and older. Change in back VAS pain from baseline to each follow-up time point was significant (*p* < 0.0001) in all populations

**Fig. 4:**
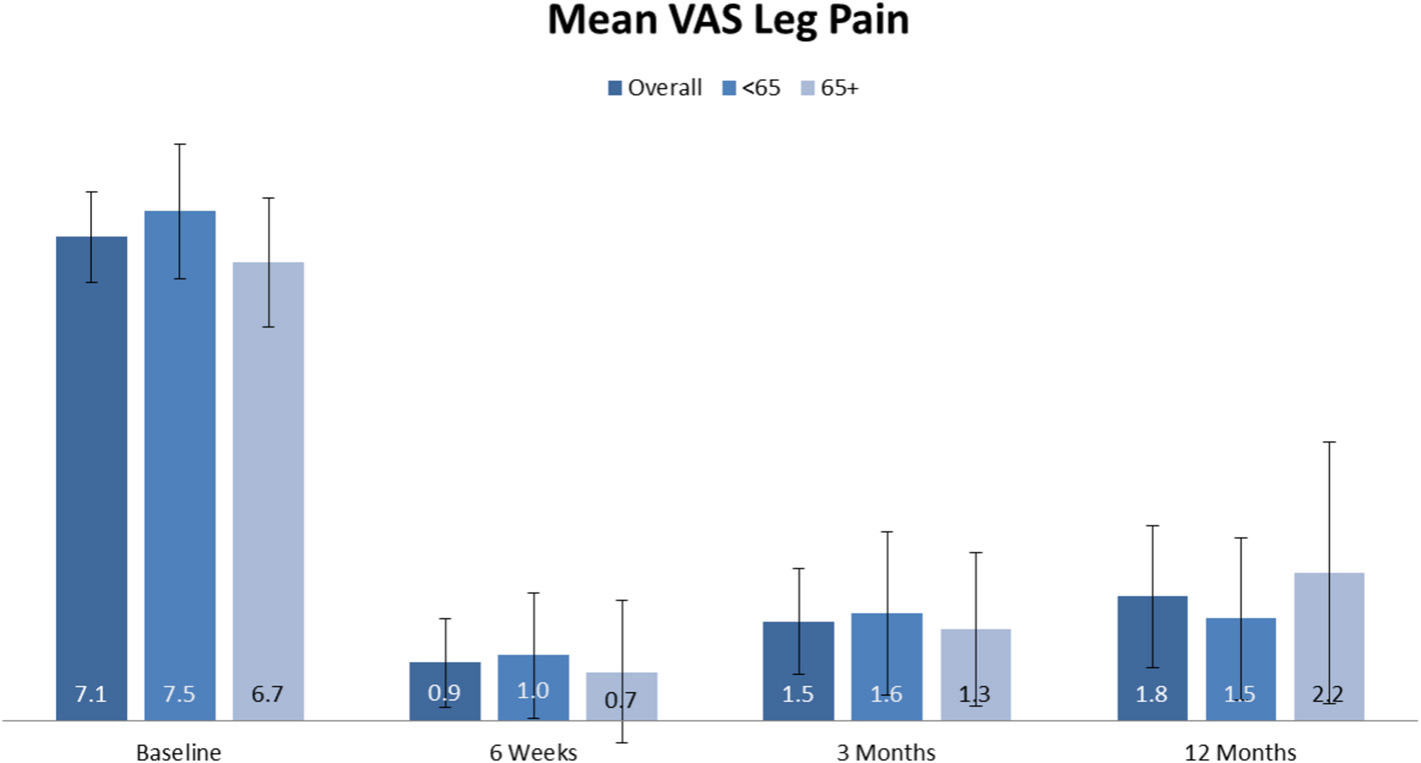
Leg VAS pain scores at baseline, 2 weeks, and at 3 and 12 months. At each time-point, the *bars* from *left* to *right* indicate scores for the entire population, subjects aged less than 65 years old, and those 65 years and older. Change in leg VAS pain from baseline to each follow-up time point was significant (*p* < 0.0001) in all populations

There were statistically significant improvements in both leg and back pain outcomes after surgery. Compared with the preoperative leg or back pain level, the postoperative pain levels were significantly (*p* < 0.0001) improved at every time point (Figs. 3 and 4). The mean improvement in the leg pain VAS scores from baseline to 2 weeks and 3 and 12 months was 6.2, 5.5, and 5.3 cm, respectively. For back pain, the mean improvement in VAS scores from baseline to 2 weeks and 3 and 12 months was 3.9, 4.3, and 4.2 cm, respectively. A comparison between subjects greater than 65 years old and those less than 65 years old revealed no significant differences for either leg or back pain at any time point.

One subject (2.3 %) exhibited a neurological deficit (worsening leg pain) at 3 months, which was resolved by 12 months.

### Safety

There were no adverse events that were related to TE/autograft. There were no infections attributed to the graft material, and there were no deep infections. Two subjects (4.7 %) had superficial infections that were treated with irrigation, debridement, and antibiotics without sequelae.

There were three revision surgeries (7 %). Dislodged hardware caused one revision, and this patient was subsequently fused at 12 months. Two were caused by adjacent level symptomatic stenosis. There were no revisions attributed to symptomatic pseudoarthrosis.

## Discussion

This retrospective, open-label study evaluated fusion and clinical outcomes for Trinity Evolution® cellular bone allograft (TE) combined with local bone in subjects who underwent decompressive lumbar laminectomy and one- or two-level posterolateral arthrodesis with supplemental pedicle screw fixation. To our knowledge, this is the first clinical report of a cellular bone allograft used for posterolateral fusions (PLF). The usage of TE did not raise any safety concerns since there were no adverse events, deep infections, or symptomatic pseudoarthroses related to TE. Subject leg and back pain significantly improved at every time point as compared to baseline with improvements that were above the minimal clinically important difference [14].

Although ICBG has been considered the gold standard for lumbar spine arthrodesis, it is associated with significant, well-documented morbidity [15–17] and potential limitations (quality and quantity). For instrumented posterolateral arthrodesis, the reported fusion rate for ICBG varied from 54 to 100 % [1, 18–24]. A comparison to one of these studies that had a similar patient population of single-level symptomatic spinal stenosis and spondylolisthesis revealed a comparable bridging bone fusion rate, but the mean angular motion was much higher (4.2° versus 1.1° in this study) [1]. From the current study, the 90.7 % fusion rate of TE used as a graft extender with locally derived autograft bone compares favorably with the reported ICBG fusion rate and mitigates the morbidity associated with ICBG harvest. In addition, TE as a graft extender was associated with fusion even in cases with low quantities of local bone.

Comorbidities such as age, tobacco use, steroid dependence, and diabetes have been linked to higher rates of non-union or delayed union and inhibition of bone repair and are associated with higher complication rates [11, 25–28]. We therefore stratified these groups out of the entire population to determine if fusion rates would be impacted. Subgroup comparison of those patients who were overweight, greater than or equal to 65 years of age, diabetic, tobacco/nicotine users, steroid dependent, and had more than one level fused showed no significant differences in fusion rates as compared to their counterparts. Since the sample size was small for several of these populations, the results must be interpreted with caution. One possibility to explain the maintenance of fusion rates even for these high-risk patient groups may be that TE mitigated the increased risk of pseudoarthrosis for these subjects.

The exception for small sample sizes was the elderly population, which contained a larger and more balanced population size than the other subgroups. Elderly patients have more overall health issues with multiple medical comorbidities that contribute to difficulty achieving successful fusion with autograft [1, 29, 30]. One potential explanation for the lack of a decrease of fusion rate that was observed between the older and younger patient subgroups is that the consistent levels of viable, healthy osteogenic cells within TE were able to compensate for the decreased cellular quantity and quality of the cells within autograft derived from elderly individuals. In order to draw any definitive conclusions, a prospective and ideally randomized, controlled study design would be required with comparisons made between high-risk and normal subject cohorts.

Published literature on cellular bone allografts supports the positive radiographic and clinical findings reported herein. In a prospective study that evaluated the safety and effectiveness of TE in single-level anterior cervical discectomy and fusion, the fusion rate was 93.5 % at 12 months, no serious allograft-related events occurred and comparisons to the literature revealed that TE may help negate any physiological barriers to fusion associated with high risk factors [31]. TE was evaluated in foot and ankle arthrodesis procedures, and fusion rates were higher than or comparable to fusion rates with autograft that were reported in the literature [12]. An earlier version of TE (Trinity Matrix) has previously been shown to be effective for healing foot fracture non-unions and avascular necrosis [32, 33], demonstrated equivalence to autograft in patient satisfaction and fusion rates [34], and showed favorable safety and effectiveness outcomes in 23 revision foot and ankle surgery procedures [35].

A limitation to this study was that TE was utilized as a graft extender with locally derived bone, and there was no control group that contained either TE or autograft alone. Additionally, six cases utilized bone stimulators as an adjunct. Thus, the sole contribution of TE in the promotion of fusion cannot be ascertained. Additionally, this was a single center study with one surgeon and thus does not capture outcomes with alternative operative approaches or fixation. Furthermore, the surgeon evaluated that the fusion status and surgeon bias is known with respect to evaluation of other clinical outcome parameters. This potential bias was mitigated by a fusion definition that was established prior to subject enrollment and was required to be met independently of clinical outcome. Reduction of bias was demonstrated upon review of several case charts in which the surgeon originally recorded the subject as fused and the subject doing well during routine clinical follow-up, but upon re-reading of the films during the study and performed independently of outcome, the fusion status was reversed to radiographic pseudoarthrosis. Additionally, although fusion was assessed using plain films in combination with motion, the surgeon’s standard of care did not utilize thin cut computed tomography (CT). CT can increase the specificity of observing bridging bone and may also decrease the false positive rate. Lastly, the retrospective design has inherent shortcomings that included subjects that may not return within the 12-month visit window. Most subjects who did not return for their 12-month visit had early favorable outcomes. Retrospective designs may have few standardized clinical outcome measures since they are usually subject to standard of care practices. Although this study did collect pain information in the form of patient-generated lower back and leg VAS questionnaires, more comprehensive functional measures such as Oswestry Disability Index were not part of this clinician’s standard practice.

## Conclusions

In conclusion, the results of this clinical study suggest that TE combined with local bone achieves high rates of fusion as compared to the literature in a broad patient population, including those at high risk for complications and older patients representative of the Medicare population. TE possesses the osteoconductive, osteoinductive, and osteogenic biological properties of autograft bone, while avoiding donor site morbidity and limited quantity issues of iliac crest autograft as well as additional operating room time associated with harvesting autogenous iliac crest bone. Therefore, TE potentially provides important advantages to both the patient and the surgeon. In addition, since TE is exclusively derived from donors that undergo a very strict screening process and each lot is tested to meet specific criteria regarding cell viability and BMP-2 content, the potential decreased potency of autograft derived from older or high-risk patients may be avoided when TE is utilized as an extender. Appropriately powered randomized controlled studies with longer follow-up times that compare TE to autograft are warranted in the future.

## Abbreviations

A/P: anterior/posterior
BGS: bone graft substitute
BMI: body mass index
BMP: bone morphogenetic protein
CT: computed tomography
DBM: demineralized bone matrices
DDD: degenerative disc disease
ICBG: iliac crest bone graft
PLF: posterolateral fusions
SD: standard deviation
TE: Trinity Evolution® cellular bone allograft
VAS: visual analogue scale
